# Over-expression of gluconic acid in *Aspergillus oryzae* RP-21 mutants generated by a random mutagenesis approach

**DOI:** 10.1007/s13205-012-0049-5

**Published:** 2012-03-02

**Authors:** Sunhare Raksha, Sharmila Srinivasan, Garima Prasant, Rajagopalan Prabu

**Affiliations:** 1 Institute of Bioscience, Universiti Putra Malaysia, 43400 UPM Serdang, Selangor Malaysia; 2 School of Chemical and Biotechnology, SASTRA University, Thanjavur, 613401 India; 3 Amity Institute of Biotechnology, Jaipur, 302006 India; 4 Department of Bioprocess Technology, Faculty of Biotechnology and Biomolecular Sciences, Universiti Putra Malaysia, 43400 UPM Serdang, Selangor Malaysia

**Keywords:** *Aspergillus. oryzae*, *N*-Methyl-*N*′ nitro-*N*-nitrosoguanidine **(**NTG), Mutation, Gluconic acid

## Abstract

Random mutagenesis with *N*-methyl-*N*′ nitro-*N*-nitrosoguanidine (NTG) was used to mutate *Aspergillus oryzae* RP-21 to develop high gluconic acid-producing mutants. Forty mutant colonies (designated as *A. oryzae* strains RP-NTG-01 to RP-NTG-40) screened for gluconic acid, glucose dehydrogenase and glucose oxidase production using a 12-well plate method showed that 17 strains (positive mutants) produced high concentrations of these three products, whereas 12 strains (negative mutants) showed low concentrations and the remaining 11 strains (non-mutants) did not produce any of the three products. Detailed studies of *A*. *oryzae* RP-NTG-12, a positive mutant, produced gluconic acid of up to 72 g/L in batch fermentation, which was a 2.4-fold increase in yield to that of the strain and as expected it also possessed higher activities of cell-bound glucose dehydrogenase and glucose oxidase, key enzymes of the multi-functional gluconic acid synthesis pathway. We discuss changes in the cell-bound enzyme activities of the mutants and the wild type and speculate on a mechanism for this increase. The mutant strain, *A*. *oryzae* RP-NTG-12, and the random mutagenesis method used to increase bioproducts have a good potential for developing fermentation processes to an industrial scale as demonstrated by this study.

## Introduction

Though gluconic acid (pentahydroxycaproic acid) and its salts have many applications in the food, medical, beverage and textile industries (Lee et al. 1996), its one of the more outstanding applications is use in solubilization of phosphate and as an additive for enhancing the resistance and stability of cement under extreme climatic conditions in the construction industry (Anastassiadis and Rehm 2006). Gluconic acid or sodium gluconate can be produced by chemical, electrochemical, biochemical and bioelectrochemical methods (Lee et al. 1999). Due to the high production costs and low yields by these processes, its commercialization has not been successfully achieved (Lee et al. 1999). The current preferred economical production of gluconic acid is by submerged fermentation with *Aspergillus niger,* in which glucose is converted at a rate of 15 g/L/h. This process involves fed-batch cultivation, in which glucose is added intermittently at 34 °C and the pH is maintained at 6.0–6.5 neutralization by sodium hydroxide. Several variations to this process have also been described (Lee et al. 1998). In addition, gluconic acid-producing microorganisms, such as *Aureobasidium pullulan* (Anastassiadis and Rehm 2006), *Pseudomonas fluorescens* (Werra et al. 2009), *Aspergillus terreus* (Dowdells et al. 2010) and *Enterobacter intermedium* (Gomez et al. 2010) have also been isolated. However, the results of gluconic acid production have met with mixed results. An alternate approach to the isolation of new strains is to mutate a promising strain and screen for mutants that are better producers (Prabu et al. 2011). *N*-methyl-*N*′-nitro-*N*-nitrosoguanidine (NTG) and ultraviolet (UV) radiation have been successfully used to enhance the industrial production of lipase (Bapiraju et al. 2004), xylitol (Rao et al. 2006), L-DOPA (Haq and Ali 2006) and kojic acid (Prabu et al. 2011). Prabu et al. (2011) have suggested that random mutagenesis approach for industrial strain improvement is simple, easy and effective when compared with recombination technologies and have therefore advocated this approach for generating mutants. The aim of the study reported here was to use random mutagenesis on the wild-type *A*. *oryzae* and generate mutants with improved gluconic acid yields, and to evaluate the performance of these mutants in terms of the yield of gluconic produced per carbon of glucose consumed in shake flask experiments.

## Materials and methods

**Fig. 1:**
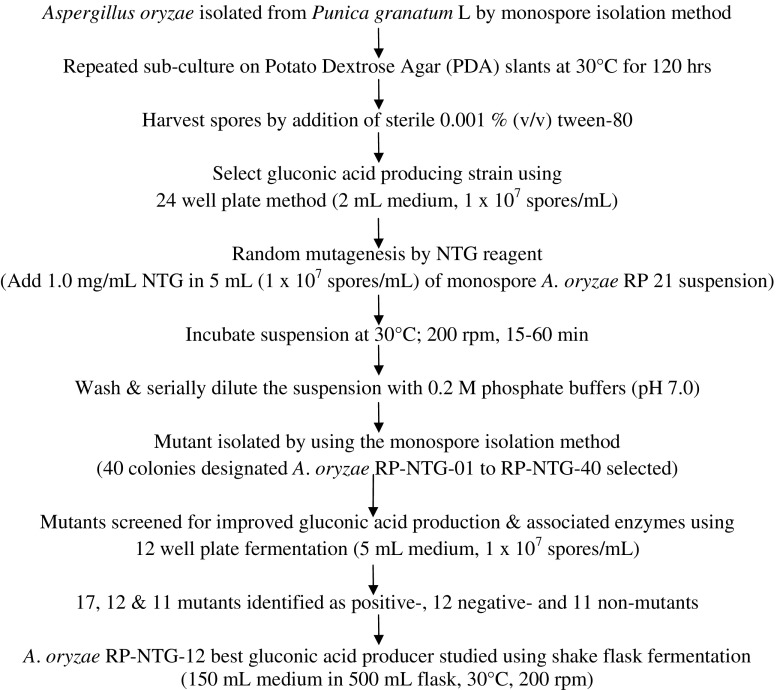
A summary of the steps, procedures and outcomes on the random mutagenesis studies of *Aspergillus niger* RP-21

The steps involved in generating mutants from the parent wild-type strain *A. niger* RP-2 by random mutagenesis*,* screening of high gluconic acid-producing mutants and studies on the enzymes of the gluconic acid pathway are illustrated in Fig. 1.

### Microorganism

The parent wild-type strain *A. oryzae* RP-21 was isolated from infected pomegranate pollen (*Punica granatum* L) by using the monospore isolation method as reported previously (Prabu et al. 2011).

### Mutation and isolation of high-level gluconic acid-producing mutants

Monospores were produced by repeated sub-culturing of *A. oryzae* RP-21 on Potato Dextrose Agar (PDA) slants at 30 °C for 120 h. Initially, 20–30 single colonies were isolated and sub-cultured for monospores. Sterile 0.001% (v/v) Tween-80 was added and the slants shaken for 5 min in order to harvest the spores. The harvested spores were then washed three times with 0.5 M phosphate buffer (pH 7.0), the spore suspension serially diluted with phosphate buffer and sub-cultured onto PDA agar plates for the isolation of single colonies. The standardized spore suspension (approximately 1 × 10^7^ spores/mL medium) was used to screen for gluconic acid production using the 24-well plate method (2 mL) and/or shake flask cultures (150 mL). The highest gluconic acid-producing culture derived from the monospore suspension was selected and mutated with *N*-methyl-*N*′ nitro-*N*-nitrosoguanidine **(**NTG) as described by Prabu et al. (2011). One mL of NTG (1.0 mg/mL) was added to 5 mL of the gluconic acid-producing monospore suspension (1 × 10^7^ spores/mL) in a 50-mL sterile Falcon tube. The mixture was incubated at 30 °C in a rotary shaker, agitated at 200 rpm for different time intervals (15, 30, 45 and 60 min), washed three times with 0.2 M phosphate buffer (pH 7.0), subsequently serially diluted with the same buffer and sub-cultured onto PDA agar plates. Single well-isolated colonies that developed were selected and further sub-cultured onto solid PDA agar plates with the conditions described above.

### Fermentation and extraction of enzymes from mycelia

Forty single well-isolated colonies (designated *A*. *oryzae* RP-NTG-01 to RP-NTG-40) were selected by the monospore isolation method and used to determine their ability to produce gluconic acid. Spore suspensions (1 × 10^7^ spores/mL) of *A*. *oryzae* mutant strains designated RP-NTG-01 to RP-NTG-40 and the parent wild type were initially screened for gluconic acid production using a 12-well plate fermentation method. In this method, 5 mL of medium [(g/L) glucose 120; yeast extract 40; KH_2_PO_4_ 1.0; K_2_HPO_4_ 1.0; MgSO_4_ 0.5; NaCl 1.5; Na SO_4_ 1.5; pH 4.0] was inoculated with 0.1 mL of spore suspension and incubated at 30 °C for 240 h. Subsequently, the best gluconic acid producer was tested further using batch fermentation in 500-mL shake flasks containing 150 mL of medium. In this case, the flasks were inoculated with 1.5 mL spore suspension (1 × 10^7^ spores/mL), incubated at 30 °C on a rotary shaker and agitated at 200 rpm to initiate fermentation. The fermentation broth was filtered through a vacuum filter at 4 °C, the mycelia washed two to three times with an equal amount of phosphate buffer (pH 7, 0.01 M) and the weight of the cell paste determined. Then, 20 mL of ice-chilled phosphate buffer (pH 7.2, 0.1 M) was added to approximately 20 g f mycelia and the cells lysed by ultrasonication (Elyas et al. 2010). The homogenate was centrifuged (Kubota model 2010) at 4,000×*g* for 10 min at 4 °C and the clear supernatant was used to determine glucose, gluconic acid, glucose oxidase and glucose dehydrogenase.

### Analytical methods

The concentration of gluconic acid was estimated using an HPLC (Lee et al. 1999), glucose concentration was determined by dinitrosalicylic acid (DNS) as described by Miller et al. 1960), glucose dehydrogenase activity was assayed as per the method of Lamble et al. (2003), and glucose oxidase enzyme activity was assayed according to Bergmeyer et al. (1974).

## Results and discussion

Initial screening of the 40 mutants of *A*. *oryzae* RP-21 (designated *A. oryzae* strains RP-NTG-01 to RP-NTG-40) using the 12-well plate method showed that 17 strains (positive mutants) produced high concentrations of gluconic acid, 12 strains (negative mutants) showed poor gluconic acid production and the remaining 11 strains (non-mutants) were unable to produce gluconic acid. These results demonstrate that random mutagenesis using NTG can be used to improve the ability of *A. oryzae* RP-21 to produce gluconic acid. NTG stimulates a relatively wide spectrum of mutations by alkylating pyrimidines and purines, of which the G:C → A:T transition is more prevalent than the A:T → G:C transitions (Prabu et al. 2011). This type of mutation could damage the genome of the gluconic acid-producing fungus (*A. oryzae* RP-21) and hence alter the gluconic acid synthesis enzymes at a transcription and/or translation level. Such alterations could lead to an increase in enzyme production or improve the active site for the binding of the substrate for conversion to the target metabolite (Gunka et al. 2010).

Of the 17 strains of positive mutants, *A*. *oryzae* RP-NTG-12 was the highest gluconic acid producer and hence this strain was tested further. A comparison of the positive mutant *A*. *oryzae* RP-NTG-12, the negative mutant *A*. *oryzae* RP-NTG-31 and the wild-type *A. oryzae* RP-21 is shown in Table 1. The results demonstrate that two enzymes, namely glucose dehydrogenase and glucose oxidase, had higher activities in the positive mutant in comparison to the negative mutant or the wild-type strain and it also produced higher concentrations (2.4-fold) of gluconic acid. In addition, a number of other positive mutants that had been assayed also showed elevated levels (1.6- to 1.8-fold) of these two enzymes (data not shown). This would suggest that these two enzymes could play an important role in gluconic acid synthesis.

**Table 1:** Performance of gluconic acid (GA) producing mutants and the parent strain

*A. niger* strain	Fermentation time (h)	Maximum biomass. (g/L)	Maximum GA conc. (g/L)	GA^a^ yield based on glucose consumed (g/g)	Cell yield based on glucose consumed (g/g)	GA^a^ based on cell mass (g/g)	Glucose dehydrogenase activity (U/mL)	Glucose oxidase activity (U/mL)
RP-21 (parent strain)	432	20 ± 0.8	30 ± 0.5	0.33 ± 0.01	0.66 ± 0.02	1.5 ± 0.8	8.9 ± 0.5	3.6 ± 0.2
RP-NTG-12 (positive mutant)	432	20 ± 0.6	72 ± 0.6	0.65 ± 0.03	0.18 ± 0.02	3.6 ± 0.6	22 ± 0.8	9.1 ± 0.3
RP-NTG-31 (negative mutant)	500	22 ± 0.6	10 ± 0.5	0.14 ± 0.02	0.3 ± 0.005	0.45 ± 0.6	3.1 ± 0.3	1.2 ± 0.1

^a^Gluconic acid

**Fig. 2:**
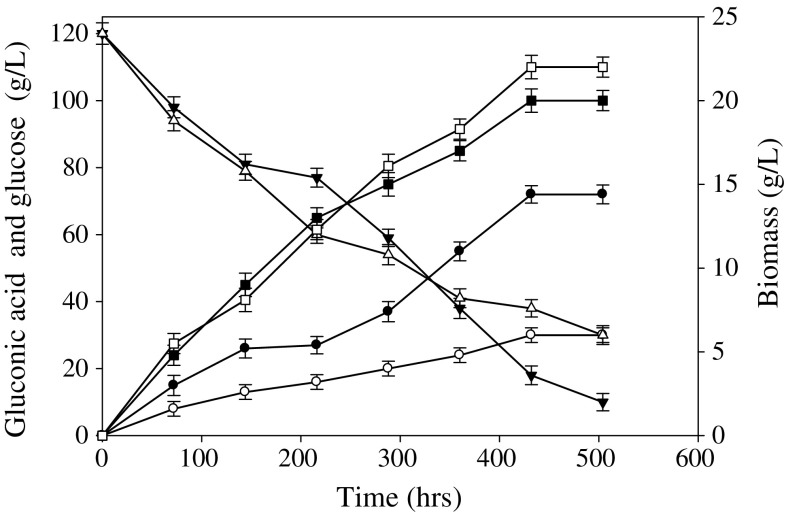
Time course of the growth of the positive mutant *A*. *oryzae* RP-NTG-12 with respect to biomass production (*filled squares*), glucose utilization (*filled inverted triangles*) and gluconic acid production (*filled circles*) in comparison to that of the wild-type *A. oryzae* RP-21’s biomass production (*open squares*), glucose utilization (*open triangles*) and gluconic acid production (*open circles*)

The time course study for gluconic acid production in batch fermentation by the positive mutant *A*. *oryzae* RP-NTG-12 and the wild-type *A*. *oryzae* RP21 is shown in Fig. 2. During exponential growth of the positive mutant *A*. *oryzae* RP-NTG-12, the concentration of gluconic acid increased with a concomitant decrease in glucose concentration. This suggested that glucose was converted to gluconic acid. The maximum concentration of gluconic acid (72 ± 0.6 g/L) was achieved after about 432 h of incubation. There was no further increase in gluconic acid production in the stationary phase, even though some glucose still remained. This result demonstrated that the non-growing mycelia were unable to convert glucose into gluconic acid, and therefore active growth was necessary for the conversion of glucose to gluconic acid. In contrast, the wild-type *A. oryzae* RP21 produced the same amount of biomass, but only 10 ± 0.5 g/L gluconic acid even after consuming 70% of the total glucose, suggesting that the glucose was converted into biomass and not gluconic acid. Similarly, the stationary phase mycelium was unable to convert the glucose to gluconic acid.

**Fig. 3:**
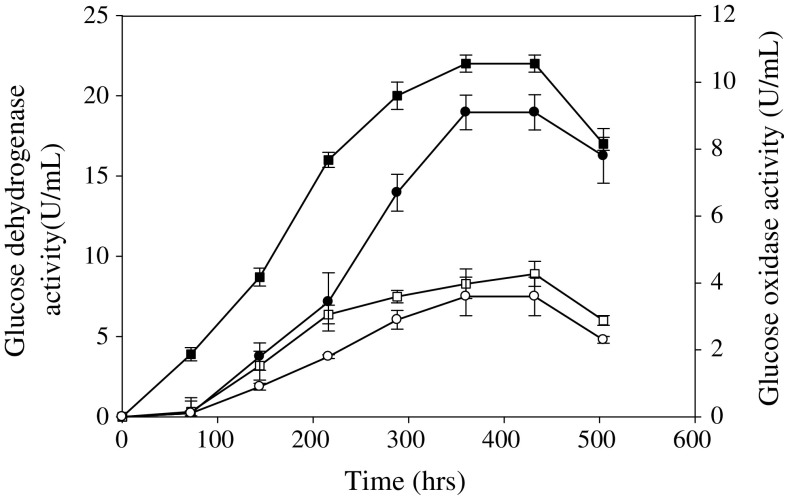
Time course of the positive mutant *A*. *oryzae* RP-NTG-12 with respect to the production of glucose oxidase (*filled circles*) and glucose dehydrogenase (*open squares*) activities in comparison to that of the wild-type *A. oryzae* RP-21’s glucose oxidase (*open circles*) and glucose dehydrogenase (*filled squares*) activities

The profiles of the two enzymes, glucose dehydrogenase and glucose oxidase, for the positive mutant *A*. *oryzae* RP-NTG-12 and the negative mutant *A*. *oryzae* RP-NTG-31 during growth is shown in Fig. 3. The concentration of glucose dehydrogenase was the highest (20 ± 0.8 U/mL) between 350 and 450 h with the positive mutant *A*. *oryzae* RP-NTG-12 and was 2.4-fold higher than that of the wild-type strain (8.9 ± 0.5 U/mL). Similarly, the glucose oxidase concentration (9.1 ± 0.3 U/mL) peaked between 350 and 450 h and was also approximately 2.5-fold higher than the strain (3.1 ± 0.3 U/mL). Higher concentrations of glucose dehydrogenase and glucose oxidase during fermentation with the positive mutant, *A*. *oryzae* RP-NTG-12, as compared to the strain is linked to the production of the higher concentrations of gluconic acid. In addition, the negative mutant, *A*. *oryzae* RP-NTG-31, had low concentrations of glucose dehydrogenase and glucose oxidase and also produced low concentrations of gluconic acid as compared to the strain, while the cell mass remained the same. We therefore speculate that the suppressor gene of the enzymes involved in gluconic acid synthesis in the positive mutant *A*. *oryzae* RP-NTG-12 could have been mutated either by a frame shift mutation producing an inactive protein or by a base change (substitution) which could lead to a change in the amino acid composition and, therefore, a change in the conformation of this protein. This would result in the suppressor protein not being able to regulate the feedback mechanism of the enzymes involved in gluconic acid synthesis and thereby resulting in higher enzyme secretion. More work will need to be undertaken to determine the basis of the improvement in gluconic acid production by the positive mutant *A. oryzae* RP-NTG-12.

This simple approach of random mutagenesis to improve industrial strain development process has more advantages as compared to fermentation optimization strategies (Cheema et al. 2002), which usually involve a multitude of steps such as use of cheaper carbohydrate sources (Singh and Singh 2006), feedback regulation by pH adjustments (Dowdells et al. 2010), the use of fungal spores as biocatalysts (Ramachandran et al. 2007) and solid-state fermentation (Sharma et al. 2008).

## Conclusion

Our study has demonstrated that random mutagenesis with NTG can be successfully used to improve gluconic acid production. The positive mutant, *A. oryzae* RP-NTG-12, derived from random mutagenesis, was capable of producing gluconic acid up to a final concentration of 72 g/L in shake flask batch fermentation. This type of strain development is simple, reproducible and can be useful for the development of fermentation process for any industrial product.
